# Risk factors, microbiology and management of infected lymphocyst after lymphadenectomy for gynecologic malignancies

**DOI:** 10.1007/s00404-018-4914-2

**Published:** 2018-09-29

**Authors:** Xuegong Ma, Yingmei Wang, Aiping Fan, Mengting Dong, Xin Zhao, Xuhong Zhang, Fengxia Xue

**Affiliations:** 1 Department of Gynecology and Obstetrics, Tianjin Medical University General Hospital, 154# AnShan Road, Heping District, Tianjin, 300052 People’s Republic of China; 2 Department of Radiology, Tianjin Medical University General Hospital, 154# AnShan Road, Heping District, Tianjin, 300052 People’s Republic of China

**Keywords:** Infected lymphocele, Risk factor, Microbiology, Percutaneous catheter drainage

## Abstract

**Objective:**

To evaluate risk factors, microbiology and management of infected lymphocysts in patients undergoing systemic lymphadenectomy for gynecological cancer.

**Methods:**

Patients with gynecological cancer who developed postoperative lymphocysts after lymphadenectomy were enrolled between January 2009 and June 2017. The clinical data of infected lymphocysts were analyzed and compared with non-infected lymphocysts. Multivariate analysis of risk factors, the microbiology and therapeutic strategies for infected lymphocysts were also evaluated.

**Results:**

A total of 115 patients out of 619 developed postoperative lymphocysts, the incidence of infected lymphocysts was 4.36%. Infected lymphocysts were more frequently found in patients with combined pelvic and para aortic lymphadenectomy, higher number of resected pelvic lymph nodes, lower level of postoperative serum hemoglobin and higher proportion of neutropenia. The median diameter of infected lymphocysts was significantly larger than non-infected (71.89 vs 38.47 mm, *P* < 0.001) and a large size (diameter over 60 mm) was identified as an independent risk factor for infected lymphocysts (OR = 3.933, *P* = 0.017). The microbiology of infected lymphocysts includes gram-positive cocci, gram-negative bacillus and anaerobic bacteria. Percutaneous catheter drainage was successfully performed in 20 patients with infected lymphocysts. 16 of 19 patients with large lymphoceles received combined antibiobics and PCD therapy and showed clinical remission in all cases. Patients with large size infected lymphocysts who received combined therapy experienced a significantly shorter treatment period and lower recurrent rate than those with only antibiotics (*P* = 0.046, *P* = 0.018).

**Conclusions:**

The current study demonstrated that a diameter over 60 mm was an independent risk factor for infected lymphocysts. The predominant bacteria originated from the urogenital or skin flora. The combination of PCD with appropriate antibiotics was a convenient and effective therapeutic strategy resulting in a high success rate.

## Introduction

Lymphocyst, also known as lymphocele, is a cystic collection of lymphatic fluid appearing after systematic pelvic and/or para-aortic lymphadenectomy [1]. Retroperitoneal lymphadenectomy is a main component of staging and debulking surgery for gynecological malignancies including endometrial, ovarian and cervical cancers. Lymphoceles are frequently formed by the collection of lymphatic fluid from lymphatic capillaries into the retroperitoneal space. These clinical complications were observed and verified by Mori and Ferguson using contrast agents in lymphatic vessels [2].

The incidence of lymphocele following lymphadenectomy treatment in patients with gynecological malignancies ranged from 1 to 58.5% based on different studies [3]. The majority of lymphoceles were asymptomatic and found by ultrasonic or computed tomography imaging during routine postoperative follow-up. About 5–34.5% of lymphoceles are in large size and symptomatic causing excessive pressure to adjacent tissues or because of infection complications [4].

Infected lymphocele, a common complication of postoperative lymphocele, is caused by bacterial infection via the lymphatic, haematogenous or local dissemination. Lymphocele infection is the main cause of postoperative morbidity in gynecological malignant patients. It not only induces severe complications such as deep vein thrombosis (DVT), hydronephrosis, sepsis and lower extremity edema but also delays postoperative adjuvant treatment including chemotherapy and radiotherapy.

Risk factors and microorganisms that contribute to infected lymphoceles are still to be deciphered. The aim of this study was to identify the risk factors for infected lymphocele formation and to investigate the microbiology and management of infected lymphocele.

## Materials and methods

**Fig. 1:**
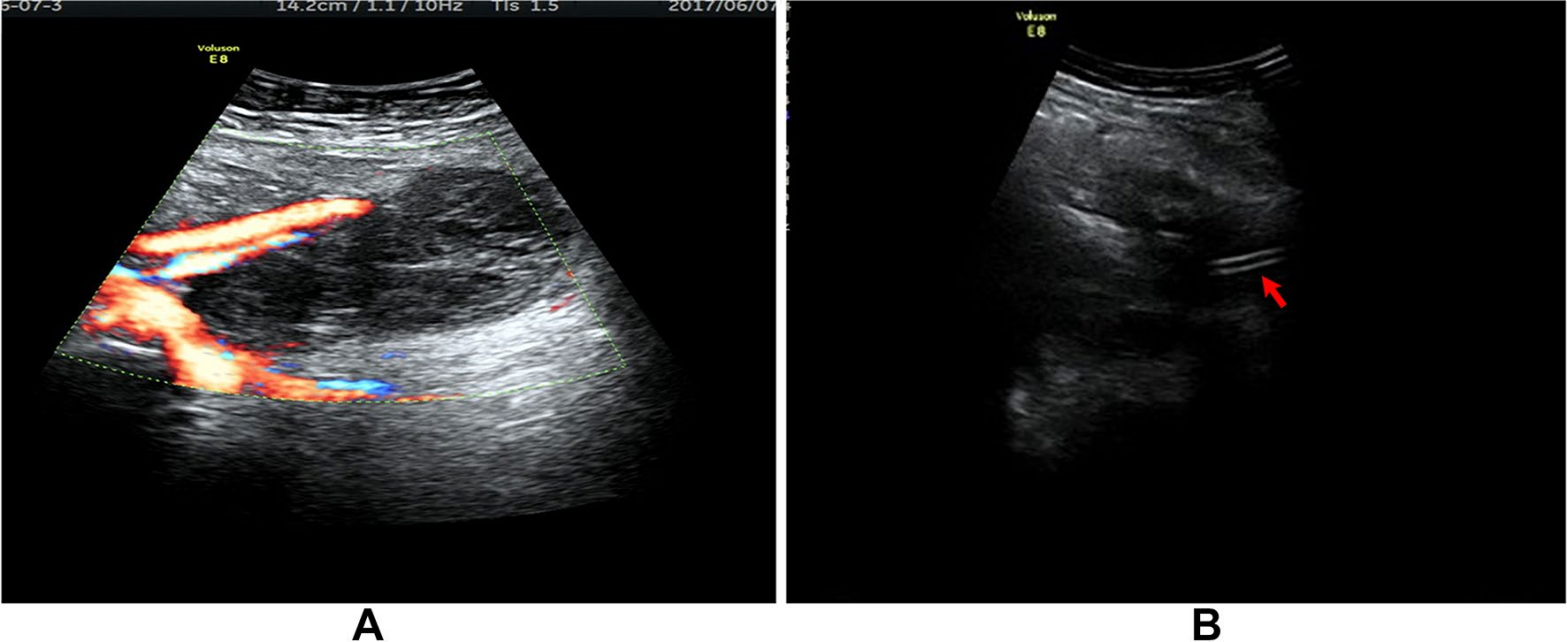
Ultrasonography showed thick-walled cyst with turbid content in mixed acho along the pelvic wall surrounded by iliac blood vessels (**a**); diminished lymphocele after percutaneous catheter drainage (red arrow: catheter) (**b**)

**Fig. 2:**
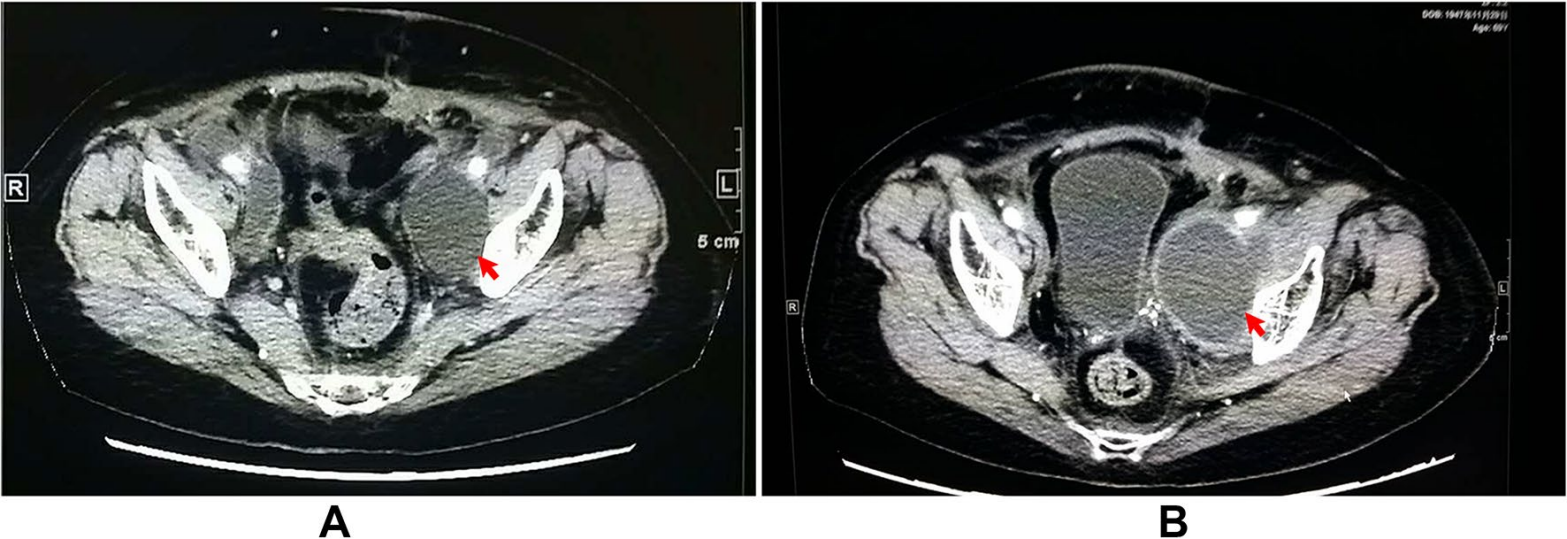
A lymphocele scanned by CT revealing a smooth and thin-walled cavity filled in water-equivalent fluid (**a**); the same lymphocele became infected and showed low-attenuation with thickened enhanced wall in the same position (**b**)

**Fig. 3:**
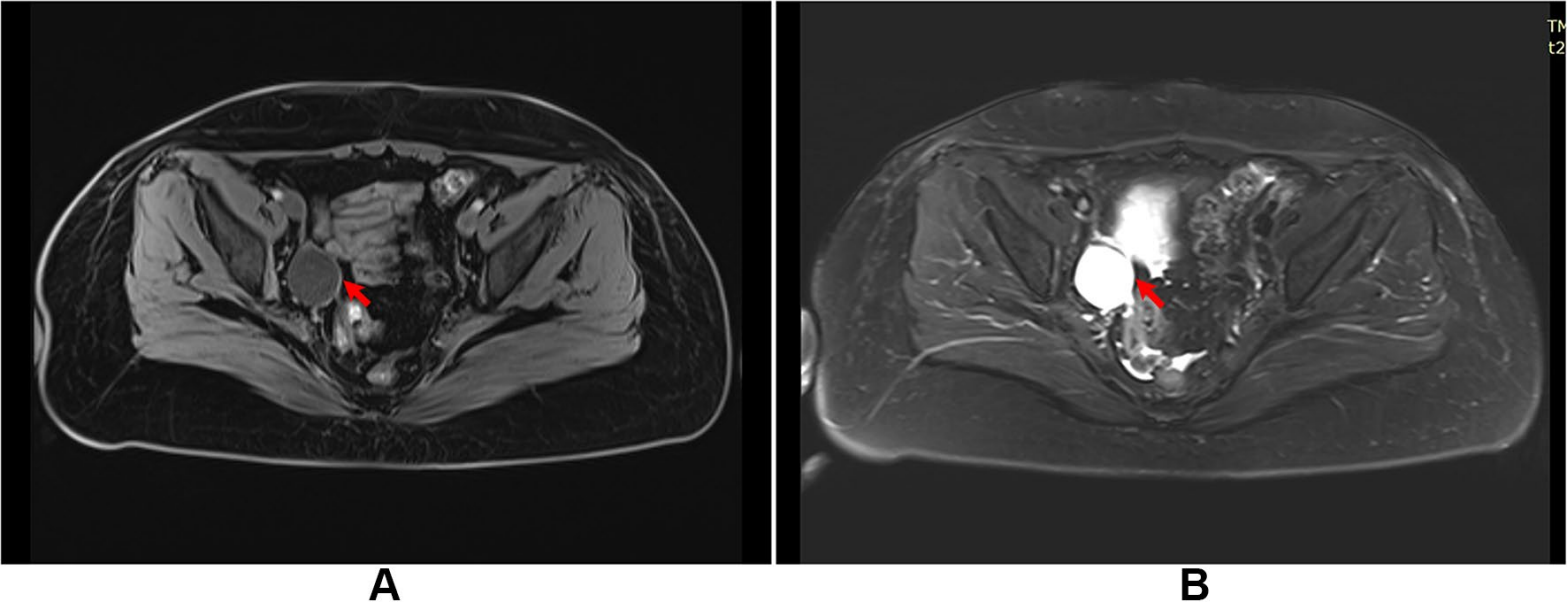
An infected lymphocele scanned by MRI showing hypo-intense T1-weighted (**a**) and hyper-intense signals on T2-weighted images (**b**)

A retrospective study was carried out on 619 gynecologic cancer patients who received pelvic and/or combined para aortic lymphadenectomy as a primary surgical staging treatment or debulking surgery at Tianjin Medical University General Hospital between January 2009 and June 2017. All surgical procedures were performed either through laparotomy or laparoscopy. Pelvic lymph nodes were resected from the external iliac, common iliac, obturator to interiliac regions by monopolar, bipolar coagulation and ultrasonic scalpel. For patients who received para aortic lymphadenectomy, the lymphatic tissues were removed from the para aortic and para caval region up to the inferior mesenteric artery or to the level of the renal veins. The retroperitoneum was left open or closed after surgery and pelvic drains were placed retroperitoneally for managing intraoperative bleeding or exudation. The drains were removed when the fluid drainage was less than 50 ml after 24 h. All patients received intensive follow-up care and monitoring after primary surgery. Routine physical examinations including a bimanual pelvic examination, vaginal vault cytology and transabdominal or transvaginal ultrasonography was performed during follow-up. Lymphocele was detected by clinical examination and imaging, which included ultrasonography, computed tomography (CT) and magnetic resonance imaging (MRI). The ultrasonic images of lymphoceles appeared as thin-walled pelvic cysts with clear margins arising from the pelvic wall surrounded by iliac blood vessels. CT scans revealed smooth and thin-walled cavities filled in water-equivalent fluid with no sign of infiltration (Fig. 2a). Infected lymphocele were diagnosed using the following criteria: a fever higher than 38 °C over 24 h since lymphadenectomy; pelvic pain or lower abdominal tenderness; laboratory data indicating leukocytosis or elevated C-reactive protein; and purulent fluid from percutaneous drainage. Ultrasonography showed thick-walled cysts with turbid contents in mixed acho along the pelvic wall surrounded by iliac blood vessels (Fig. 1); CT scans showed low-attenuation lesions with a thickened enhanced wall in the pelvic spaces (Fig. 2b); the infected lymphocele was observed by MRI as hyper-intense signals on T2-weighted and hypo-intense T1-weighted images with an enhancing wall (Fig. 3).

Infected lymphocele fluid specimens were obtained by ultrasonography or CT-guided aspiration and percutaneous drainage for microorganism culture. All lymphocele fluid and blood cultures for the evaluation of bacterial spectrum were performed by the microbiology laboratory at Tianjin Medical University General Hospital.

### Statistical analysis

The software SPSS Statistics 20.0 (IBM Corporation) was used for statistical analysis. Continuous variables were presented as arithmetic mean and standard deviations (SD), with median and minimum–maximum ranges. Difference of data between infected and non-infected group were analyzed using the *t*, the Chi squared and Fisher’s exact tests. The multivariate analysis was performed using logistic regression analysis. The odds ratios (ORs) with corresponding 95% confidence intervals were calculated and a value of *P* < 0.05 was considered statistically significant for all analyses.

## Results

During the study period, a total of 115 patients out of 619 developed postoperative lymphocysts, among which, infected lymphocele occurred in 27 patients. The incidence of infected lymphocele was 4.36% for gynecologic cancer patients who received retroperitoneal lymphadenectomy in this study. The median time from surgery to the diagnosis of infected lymphocele was 74 days (range 5–710 days), and was significantly longer than non-infected lymphocele (35 days, *P* = 0.008). Of the 27 infected lymphoceles, 11 (40.7%) were located on the left side of the pelvic region, 4 (14.8%) on the right side and 12 patients (44.4%) had bilateral lymphoceles. The median diameter of the infected lymphocele was 71.89 mm, which was significantly larger than non-infected lymphocele (38.47 mm, *P* < 0.001). Most patients with infected lymphocele had more than one symptom. 25 of the 27 patients suffered from mild to severe fever (37.6–40 °C). 13 patients complained of lower abdominal pain or distension and two patients developed edema of the lower limb.

The clinical characteristics of both infected and non-infected lymphoceles were analyzed in the study to evaluate the possible risk factors for infected lymphoceles (Table 1). There was no significant difference in median age and BMI between the two groups and no significant difference in cancer types between two groups (*P* = 0.281). The laparoscopic approach was performed in 12 patients (44.4%) and radical hysterectomy in 8 (29.6%) patients in the infected lymphocele group, which was similar to the non-infected group (60.2%, 28.7%). A total of 21(77.8%) patients with infected lymphoceles received combined pelvic and para aortic lymphadenectomy. The frequency of combined pelvic and para aortic lymphadenectomy was significantly higher in the infected lymphocele patients compared to non-infected (77.8% vs 22.2%, *P* = 0.024). The median number of removed pelvic lymph nodes in the infected lymphocele group was significantly higher than that in non-infected group (24 vs 19, *P* = 0.027). In the infected lymphocele group, the retroperitoneum was left open during the operation in 12 patients (44.4%) and postoperative drainage was performed in 17 cases (63%). 17 of 27 (63%) patients were diagnosed with advanced FIGO stage according to postoperative pathology. No significant differences were observed in intraoperative peritoneum opening, postoperative drainage or in FIGO stage between the two groups. The proportion of patients who received postoperative radiotherapy and chemotherapy was 33.9% and 40.7%, respectively, in infected lymphocele group, which was higher than in non-infected group (18.2% and 38.6%), however, the difference was not statistically significant (*P* = 0.095, *P* = 0.845). The median hemoglobin in patients with infected lymphocele was significantly lower than non-infected group (103 vs 111 g/l, *P* = 0.004) and there was a significantly higher proportion of post-chemotherapy or radiotherapy neutropenia patients in the infected lymphocele group compared to non-infected group (48.1% vs 17.0%, *P* = 0.039). There was no significant difference in serum albumin levels between the two groups (38.3 vs 39.1, *P* = 0.531).

**Table 1:** Comparison of clinical characteristics of lymphoceles with or without infections

Characteristic	Non-infected lymphoceles (*n* = 88)	Infected lymphoceles (*n* = 27)	*P* value
Age (years)	57.15 ± 8.59	56.92 ± 9.75	0.908
BMI (kg/m^2^)	25.48 ± 4.27	25.43 ± 3.65	0.958
Hb (g/l)	111.74 ± 13.76	103.00 ± 12.29	0.004
ALB (g/l)	39.09 ± 5.65	38.33 ± 4.89	0.531
Neutropenia, *n* (%)^a^	15 (17.0%)	13 (48.1%)	0.039
Type of cancer, *n* (%)			0.281
Endometrial cancer	57 (64.8%)	21 (77.8%)	
Ovarian cancer	18 (20.5%)	2 (7.4%)	
Cervical cancer	13 (14.7%)	4 (14.8%)	
Approach, *n* (%)			0.148
Laparotomy	35 (39.8%)	15 (55.6%)	
Laparoscopy	53 (60.2%)	12 (44.4%)	
Radical hysterectomy, *n* (%)	25 (28.7%)	8 (29.6%)	0.929
Type of lymphadenectomy, *n* (%)			0.024
Sole pelvic	41 (46.6%)	6 (22.2%)	
Combined pelvic and paraaortic	47 (53.4%)	21 (77.8%)	
No. of pelvic nodes	19.08 ± 8.81	23.78 ± 11.65	0.027
No. of paraaortic nodes	6.17 ± 4.43	6.82 ± 4.68	0.580
FIGO stage, *n* (%)			0.222
I + II	66 (75.0%)	17 (63.0%)	
III + IV	22 (25.0%)	10 (37.0%)	
Retroperitoneal open	41 (46.6%)	12 (44.4%)	0.845
Postoperative drain	47 (53.4%)	17 (63.0%)	0.382
Postoperative chemotherapy	34 (38.6%)	11 (40.7%)	0.845
Postoperative radiotherapy	16 (18.2%)	9 (33.9%)	0.095
Time from surgery (day)	35.06 ± 24.69	74.40 ± 13.24	0.008
Location, *n* (%)			0.148
Bilateral	22 (25.0%)	12 (44.4%)	
Left	46 (52.3%)	11 (40.7%)	
Right	20 (22.7%)	4 (14.8%)	
Diameter of lymphocyst (mm)	38.47 ± 15.30	71.89 ± 22.85	< 0.001

*BMI* Body mass index, *Hb* haemoglobin, *ALB* albumin

^a^The value was presented as number (*n*) and percentage (%)

In multivariate analysis of risk factors for infected lymphocele, large size (diameter over 60 mm, OR = 3.933, *P* = 0.017) was identified as an independent risk factor. However, we failed to identify large number of resected pelvic lymph nodes (total number over 24, OR = 1.040, *P* = 0.151) and post-operative anemia (hemoglobin less than 110 g/l, OR = 0.980, *P* = 0.350) or neutropenia (OR = 1.534, *P* = 0.480) as well as postoperative radiotherapy (OR = 2.659, *P* = 0.101) as independent risk factors for infected lymphocele (Table 2).

**Table 2:** Multivariate analysis of risk factors for infected lymphoceles

Risk factors	OR (95%CI)	*P* value
Age	1.022 (0.957–1.092)	0.518
BMI	1.002 (0.885–1.134)	0.975
Hb < 110 g/l	0.980 (0.940–1.022)	0.350
Neutropenia	1.534 (0.468–5.028)	0.480
Radical hysterectomy	0.958 (0.307–2.989)	0.941
Combined lymphadenectomy	0.762 (0.270–2.154)	0.609
No. of pelvic nodes > 24	1.040 (0.986–1.096)	0.151
Large lymphocele^a^	3.933 (1.275–12.132)	0.017
Postoperative radiotherapy	2.659 (0.826–8.553)	0.101
Postoperative chemotherapy	0.736 (0.240–2.255)	0.592

*BMI* Body mass index, *Hb* haemoglobin, *OR* odds ratio

^a^Lymphocele with a diameter > 60 mm

Specimens of the infected lymphoceles fluid collected by ultrasound-guided catheter drainage were cultured for 19 cases and blood cultures for ten cases. There were 15 patients with monomicrobial infections and two with polymicrobial infections. Bacteria were detected in both drainage fluid and blood cultures in two of the ten cases. The most predominant cultured microorganisms present were gram-positive *Staphylococcus* species (31.6%) and gram-negative bacillus such as *Escherichia coli* (26.3%) and Enterococcus (21.0%) and other anaerobic bacteria. The microbiology of infected lymphocysts in drainage fluid and blood cultures is shown in Table 3.

**Table 3:** Microbiology of infected lymphoceles

Microbiology	Abscess culture (*n*)^a^	Blood culture (*n*)
*Escherichia coli*	5	
*Enterococcus faecalis*	4	
*Staphylococcus aureus*	2	1
*Staphylococcus lentus*	2	
*Staphylococcus epidermidis*	1	
*Staphylococcus saprophyticus*	1	1
*Klebsiella oxytoca*	1	
*Peptostreptococcus*	1	
*Streptococcus agalactiae*	1	
*Enterobacter cloacae*	1	

^a^The value was presented as number (*n*) of cases

All of 27 patients with infected lymphoceles received broad-spectrum antibiotics including cephalosporin and metronidazole, cefoperazone sulbactam or moxifloxacin, either intravenously or orally. The median duration of antibiotic treatment was 17 days (7–35 days). Percutaneous catheter drainage (PCD) therapy was performed in 20 cases with either large lymphocysts or severe symptoms, or ineffective to initial antibiotics treatment and achieved technical success in all cases. No patients experienced therapeutic complications during the PCD procedure. 16 (84.2%) of 19 patients with large lymphoceles (diameter over 60 mm) received combined antibiotics and PCD therapy and showed clinical remission in all cases. The treatment option and duration of infected lymphoceles in large size (diameter > 60 mm) or small size (diameter ≤ 60 mm) were compared in Table 4. For the patients who received only antibiotic therapy, the mean treatment period in large size group was significantly longer than the small size group (25 vs 12 days, *P* = 0.015). But the difference of treatment time between two groups who received combined therapy wan insignificant (17 vs 14 days, *P* = 0.603). Additionally, in the large size group, the patients who received combined therapy experienced a significantly shorter treatment period than those with only antibiotics (17 vs 25 days, *P* = 0.046). While there was no significant difference in treatment time between only antibiotic and combined therapy patients in the small size group. For the large infected lymphoceles, there was one case with only antibiotics therapy and one case with combined treatment recurred in 2 months and half a year, respectively. So the recurrence rate in only antibiotic subgroup was 33.3%, significantly higher than combined treatment group (6.3%, *P *= 0.018).

**Table 4:** Management of infected lymphoceles

	Large infected lymphoceles^a^ (*n* = 19)	Small infected lymphoceles^b^ (*n* = 8)	*P* value
Antibiotics only, *n* (%)	3 (15.8%)	4 (50%)	0.088
Antibiotic treatment period (day)	25 (19–35)	12 (7–17)	0.015
Antibiotics + PCD, *n* (%)	16 (84.2%)	4 (50%)	0.088
Combined treatment period (day)	17 (8–39)	14 (6–21)	0.603
Recurrence rate, *n* (%)	2(10.5%)	1 (12.5%)	0.739

*PCD* Percutaneous catheter drainage

^a^Lymphocele with a diameter > 60 mm

^b^Diameter ≤ 60 mm

## Discussion

Lymphocele formation is a common complication following lymphadenectomy for gynecological malignancies; however, infected lymphocele is uncommon with only a few inconsistent incidences being reported. Kawamura [5] found 13 infected lymphocysts among 878 gynecologic cancer patients and Hiramatsu [6] reported the incidence to be 2.98% in 1175 patients who underwent lymphadenectomy for gynecologic malignancies. The incidence of infected lymphocysts was 4.36% (27/619) in this current study. The formation of lymphocele is due to injury to the lymphatic vessels after lymph node dissection and excess lymphatic fluid accumulates and fills the pelvic or retroperitoneal space. A majority of lymphocysts are small and sterile, most of which are usually asymptomatic needing no additional treatment with eventually spontaneous resorption during clinic observation. However, once lymphoceles become large and infected they may compress adjacent structures and cause clinical symptoms including abdominal or pelvic pain, fever, chills, sepsis and lower limb edema [7]. Most patients with infected lymphocysts in the current study had more than one symptom, which delayed the postoperative adjuvant treatment including chemotherapy and radiotherapy. Studies have focused on the risk factors for lymphocele formation after lymphadenectomy but the potential risk factors for infected lymphoceles are yet to be deciphered.

The extent of lymph nodes dissection and the number of lymph nodes removed has been proved to increase the incidence of symptomatic lymphoceles. Zikan et al. [4] found that a total number of lymph nodes > 27 excised to be an independent risk factor for the formation of symptomatic lymphocele. In the current study, the frequency of infected lymphocele was significantly higher in patients who received combined pelvic and para aortic lymphadenectomy compared to sole pelvic lymphadenectomy. The number of pelvic lymph nodes was significantly higher in infected lymphocele group compared to the non-infected group although it was not an independent risk factor for infected lymphocele by multivariate analysis. The injury to the lymphatic vessels might be severe with extensive and a large number of lymph nodes removed. Hence, a relatively long time is needed to re-establish the lymphatic network. The permanent existence of a lymphocele allows the trends for the formation of an infected lymphocele. However, the incidence of post-operative lymphocele has been greatly reduced because of laparoscopic approaches and new technologies such as ultracision and ligaclip, which were introduced as prophylactic strategies for preventing lymphatic complications [8, 9].

Another risk factor proposed for lymphocele formation are surgical procedures including peritonealization and postoperative drainage. It has been demonstrated that peritonealization and postoperative drainage resulted in a higher risk of symptomatic lymphocele [10]. Leaving the retroperitoneum open allows the lymph to drain and be absorbed by the peritoneum and omentum hence decreasing the risk of postoperative lymphatic fluid accumulation and infection. The proportion of patients who underwent retroperitoneal opening and postoperative drainage was compared between infected lymphocele and non-infected group. However, the current study failed to find any significant differences.

We hypothesized that infected lymphocele easily manifested in the patients with poor clinical conditions such as postoperative anemia, neutropenia and hypoproteinemia. The serum levels of hemoglobin, albumin and neutrophil counts were measured in this study. The proportion of anemia and neutropenia in infected lymphocele group was significantly higher than the non-infected group. Decreased hemoglobin levels due to operative blood loss and neutropenia mainly due to postoperative chemotherapy may influence the immune function and promote inflammation. Although both anemia and neutropenia were not independent risk factors of infected lymphocele, we still believe that monitoring and maintaining the postoperative patients who received systemic lymphadenectomy in good condition is important for the prevention of lymphocele infection.

Other risk factors including age, BMI, type of cancer, surgical approach, radical hysterectomy and FIGO stage were compared between the two groups, however, no significance were found for these parameters. In a retrospective study by Kim et al. [11] found that the occurrence of lymphoceles was higher in patients with higher BMI and in patients who received postoperative radiotherapy. Another study by Zikan et al. [4] showed a significant lower incidence of symptomatic lymphoceles in patients treated with postoperative radiotherapy. They analyzed that the fibrotic reaction of the irradiated tissue and lymphatic vessels lowered the incidence of infected lymphoceles. In this study, the proportion of patients who received postoperative radiotherapy in the infected lymphocele group was higher than the non-infected group, but the difference did not reach statistical significance.

In the current study, the median time to diagnosis infected lymphocele was 74 days, significantly longer than non-infected lymphocele (35 days, *P* = 0.008). Similarly, Kawamura et al. [5] found that the median time from surgery to the onset of infection symptoms was 72 days (range 12–1120 days). While the discovery of lymphocele by physical examination or imaging before infections formation might be earlier in the infected group than non-infected because that most (70%) of the infected lymphoceles were larger in size (diameter > 60 mm). Kim et al. [9]. reported that all lymphoceles with a diameter of < 6 cm were asymptomatic, whereas lymphoceles with a diameter exceeding 6 cm were symptomatic and 69% (9/13) were infected. Similarly Kondo et al. [12] found that large lymphocyst (> 50 mm) at 3 weeks post-surgery or persistent lymphocyst increases the risk of lymphedema, lymphangitis and DVT. We found a significantly higher diameter in infected group and determined that a diameter over 60 mm as an independent risk factor for infected lymphocele (OR = 3.933, *P* = 0.017). A large lymphocele compresses adjacent tissue thereby causing congestion and establishes an environment for bacteria growth and dissemination. Due to this, a more aggressive strategy and longer treatment period for larger lymphocele is required compared to smaller one.

Antibiotic therapy has been effective to treat infected lymphocele, but the choice of antibiotics needs to be carefully selected. A few studies have demonstrated the microbiology of infected lymphocysts after lymphadenectomy in gynecologic malignancies [13, 14]. To better understand the bacterial etiology of infected lymphoceles and select effective antibiotics, both drainage fluid and blood cultures were performed in our study. The 15 (88%) patients with monomicrobial infections and 2 (12%) with polymicrobial infections were mainly caused by gram-positive cocci, gram-negative bacillus and anaerobic bacteria, most of which originated from the urogenital flora. A similar result was reported by Kawamura et al. [5] and monotherapy with either ampicillin-sulbactam or amoxicillin-clavulanic acid was recommended. Usually an empiric anti-microbial selection is made before the results of the bacterial cultures from lymphocyst fluid samples are known. From our study, a combination of antibiotics such as cephalosporin and metronidazole, which cover both gram-positive cocci, gram-negative bacillus and anaerobic bacteria is recommended. Antibiotics should be changed to cefoperazone-sulbactam or moxifloxacin based on drug sensitivity test and vancomycin should be administered for methicillin-resistant *Staphylococcus aureus* if necessary. The duration of antibiotic therapy varied in different studies and combined treatment of antibiotics and drainage were recommended because that the thickening walls of the infected lymphocele diminished local drug concentrations and prolonged treatment durations [6].

Percutaneous catheter drainage (PCD) has been considered as the first-line treatment for infected lymphocele in many studies for its convenience and efficacy. PCD is the preferred surgical procedure by many clinicians because it is safer under the guidance of ultrasonography or CT imaging, whereas surgical drainage or peritoneal marsupialization may result in surgical complications, long hospitalization and a financial burden to the patient [15]. Moreover, PCD allows flushing and persistent drainage of lymph fluids until the adhesion of the lymphocele and collateral lymphatic pathways are formulated [16]. Kim et al. [15] performed PCD under ultrasonographic guidance on 23 symptomatic or infected lymphoceles and had a successful treatment rate for all patients. Similarly Kurata et al. [16] treated ten infected lymphoceles using PCD with a 90% success rate. Hiramatsu et al. [6] found it difficult to treat infected lymphoceles by antibiotics alone in their study and advised that PCD should be introduced in addition to antibiotics for severe infections.

However, there is still no proper guidance for the indications of PCD nor appropriate timing to initiate and remove the drainage. In Kim’s research [15], PCD was performed for those with no change or increased size during antibiotic therapy or with severe symptoms. In the current study, PCD was performed in patients with infected lymphoceles with either large size (diametre over 60 mm) or severe symptoms, and those cases showing insufficient therapeutic effects to sole antibiotics treatment. In our experience, PCD concurrently combined with antibiotics significantly shortened the treatment period of large size infected lymphoceles. However, the combined therapy failed to shorten the treatment time in small size group and seemed to be unnecessary in the treatment for small infected lymphoceles. Hiramatsu et al. [6] treated severe infected lymphoceles at a median starting time of 3 days with antibiotic administration and found that starting the drainage before day 5 significantly shortened total treatment period than after day 6. In the above mentioned studies, the criteria for removal of PCD was defined as having a drainage volume less than 10 ml per day or lymphocyst decrease to a minimal size with no symptoms. The recurrence rate after PCD treatment for infected lymphoceles was 13% and 11.1% in Kim et. al’s [15] and our study, respectively. Fortunately, such a repeating procedure as reinsertion of the drainage catheter for recurrent lymphocele is still effective and makes the disease curable.

PCD with sclerotherapy has been introduced by some researches as a treatment option for postoperative lymphoceles and has a success rate of 77–100% [17–19]. The sclerosant including povidone-iodine, ethanol, tetracycline and other agents can cause an inflammatory reaction and render the lymphocele adhesive, resulting in a shorter catheter placement time. However, complications such as allergy, vascular thrombosis and nephrotoxic acute renal failure were observed [20]. Kim et al. [15] treated infected lymphoceles using PCD with or without sclerotherapy and found no significant difference between the two groups. Karcaaltincaba et al. [21] treated infected lymphoceles successfully without sclerotherapy and considered the use of sclerosing agents in infected lymphoceles as unnecessary. In addition, Kim et al. [18] regarded sclerotherapy as unnecessary for infected lymphoceles with decreasing drainage and recommended it only for those with persistent drainage volume.

In summary, infected lymphoceles occurred in 4.36% of gynecologic cancer patients who underwent post-retroperitoneal lymphadenectomy in this study. Post-operative lymphocele patients who received combined pelvic and para-aortic lymphadenectomy, with a large amount of pelvic lymph nodes resected, accompanied with post-operative anemia, neutropenia and those with large size lymphoceles were prone to lymphoceles infection. Especially the lymphocele diameter exceeding 60 mm was regarded as an independent risk factor for infected lymphocele. The microbiology of infected lymphoceles includes gram-positive cocci, gram-negative bacillus and anaerobic bacteria. Percutaneous catheter drainage was a convenient and effective therapeutic strategy resulting in a high success rate with the combination of appropriate antibiotics.
